# Deep Sequencing to Reveal Phylo-Geographic Relationships of Juquitiba Virus in Paraguay

**DOI:** 10.3390/v15091798

**Published:** 2023-08-24

**Authors:** Evans Ifebuche Nnamani, Briana Spruill-Harrell, Evan Peter Williams, Mariah K. Taylor, Robert D. Owen, Colleen B. Jonsson

**Affiliations:** 1 Department of Microbiology, Immunology, and Biochemistry, College of Medicine, University of Tennessee Health Science Center, Memphis, TN 38163, USA; innamani@uthsc.edu (E.I.N.); bspruill@uthsc.edu (B.S.-H.); ewilli99@uthsc.edu (E.P.W.); mariah.taylor1992@gmail.com (M.K.T.); 2 Centro Para El Desarrollo de Investigación Científica, Asunción C.P. 1255, Paraguay; rowen@pla.net.py; 3 Regional Biocontainment Laboratory, University of Tennessee Health Science Center, Memphis, TN 38163, USA; 4 Institute for the Study of Host-Pathogen Systems, University of Tennessee Health Science Center, Memphis, TN 38163, USA

**Keywords:** hantavirus, phylogeny, Juquitiba virus, molecular clock tree, next-generation sequencing, BEAST, phylogeography, New World

## Abstract

Several hantaviruses result in zoonotic infections of significant public health concern, causing hemorrhagic fever with renal syndrome (HFRS) or hantavirus cardiopulmonary syndrome (HCPS) in the Old and New World, respectively. Given a 35% case fatality rate, disease-causing New World hantaviruses require a greater understanding of their biology, genetic diversity, and geographical distribution. Juquitiba hantaviruses have been identified in *Oligoryzomys nigripes* in Brazil, Paraguay, and Uruguay. Brazil has reported the most HCPS cases associated with this virus. We used a multiplexed, amplicon-based PCR strategy to screen and deep-sequence the virus harbored within lung tissues collected from *Oligoryzomys* species during rodent field collections in southern (Itapúa) and western (Boquerón) Paraguay. No Juquitiba-like hantaviruses were identified in Boquerón. Herein, we report the full-length S and M segments of the Juquitiba hantaviruses identified in Paraguay from *O. nigripes*. We also report the phylogenetic relationships of the Juquitiba hantaviruses in rodents collected from Itapúa with those previously collected in Canindeyú. We showed, using the TN93 nucleotide substitution model, the coalescent (constant-size) population tree model, and Bayesian inference implemented in the Bayesian evolutionary analysis by sampling trees (BEAST) framework, that the Juquitiba virus lineage in Itapúa is distinct from that in Canindeyú. Our spatiotemporal analysis showed significantly different time to the most recent ancestor (TMRA) estimates between the M and S segments, but a common geographic origin. Our estimates suggest the additional geographic diversity of the Juquitiba virus within the Interior Atlantic Forest and highlight the need for more extensive sampling across this biome.

## 1. Introduction

Hantaviruses (genus: *Orthohantavirus;* family: *Hantaviridae*; order: *Bunyavirales*) are negative-sense, tripartite, single-stranded RNA viruses that are etiologic agents of hemorrhagic fever with renal syndrome (HFRS) in Europe and Asia and hantavirus cardiopulmonary syndrome (HCPS) in the Americas [1,2]. The first etiologic agents of hantavirus, Hantaan virus (HNTV) and Thottapalayan virus (TPMV), were isolated from *Apodemus agrarius* [3] and *Suncus murinus* in Asia [4] in 1978 and 1964, respectively. Not all hantavirus infections result in clinical symptoms, but for those that do, mortality rates range from 15% to 60% for the Old and New World hantaviruses, respectively, with no acute therapy or vaccines available [1,2]. The New World hantaviruses, which are responsible for HCPS cases, pose a significantly higher disease threat considering their case fatality rate, and numerous hantaviruses have been identified in the Americas. Given their nature as a global public health concern and potential for person-to-person transmission [5,6,7,8,9], there is a need to understand their evolution in order to gain insight into their adaptation in humans.

The New World hantaviruses are mostly harbored by rodents from the Cricetidae family. After the first outbreak of HCPS in the United States in 1993, the causative agent- Sin Nombre virus (*Orthohantavirus sinnombreense*, SNV) was identified in the deer mouse, *Peromyscus maniculatus* [10]. Subsequently, several other hantaviruses that cause HCPS have been identified in different reservoirs in the Americas. These include but are not limited to Andes virus (*Orthohantavirus andesense*, ANDV) from *Oligoryzomys longicaudatus*; Black Creek Canal virus (*Orthohantavirus nigrorivense*, BCCV) from *Sigmodon hispidus*; New York virus (NYV) from *Peromyscus leucopus*; Choclo virus (*Orthohantavirus chocloense*, CHOV) and Maporal Virus (*Orthohantavirus maporalense*, MAPV) from *Oligoryzomys fulvescens*; Rio Mamore virus (RIOMV) from *Oligoryzomys microtis*; Bayou virus (BAYV) from *Oryzomys palustris*; Laguna Negra virus (*Orthohantavirus negraense*, LANV) from *Calomys laucha*; and Juquitiba virus (JUQV) from *Oligoryzomys nigripes* [5,11,11,12,13,14,15,16,17,18,19,20,21,22,23]. In South America, clinical cases of HCPS have been reported in Argentina [24], Uruguay [25], Brazil [26], Chile [27,28], Bolivia [29], Peru [30,31], and Paraguay [32]. In Brazil, JUQV and Araraquara virus (ARAV, reservoir: Necromys lasiurus) are the two strains that have been discovered in patients [33], and about 85% of HCPS cases reported in Brazil have occurred in a region where *O. nigripes*, the reservoir of JUQV, is endemic [26,33,34,35,36,37,38,39]. The diagnosis of HPS in regions where dengue, leptospirosis, influenza, malaria, and COVID-19 are endemic remains challenging. Hence, a true assessment of the strains responsible for cases of HCPS in Brazil requires greater efforts in the sequencing of viruses from patient samples [40]. There have been three fatal cases reported that were caused by JUQV [26].

**Figure 1:**
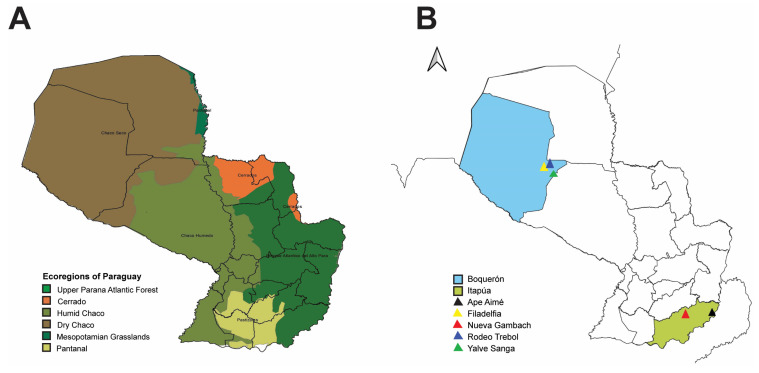
Map of Paraguay showing ecoregions of Paraguay and small mammal collection sites. (**A**) Ecoregions of Paraguay based on Clay et al. [48]. Dark green = Upper Parana Atlantic Forest, orange = Cerrado, army green = Humid Chaco, brown = Dry Chaco, hunter green = Mesopotamian Grasslands, light green = Pantanal. (**B**) Rodent collection sites in the Boquerón (blue) and Itapúa (light green) departments in Paraguay. In Boquerón, small mammals were captured in the following localities: Filadelfia (yellow), Rodeo Trebol (dark blue), and Yalve Sanga (green). In Itapúa, small mammals were captured in the localities Nueva Gambach and Ape Aimé.

Paraguay is a South American country with six ecoregions: the Dry Chaco, Humid Chaco, Pantanal, Cerrado, and Upper Paraná (Interior) Atlantic Forest and Mesopotamian Grasslands (Figure 1A) [41]. These ecoregions include tropical and subtropical regions, and the country harbors over 181 native mammal species [42,43]. Of these 181 mammalian species, 34 belong to Sigmodontinae, a subfamily of the Cricetidae family [42]. The rodents in the family Cricetidae are major reservoirs of New World hantaviruses, and the relatively well conserved natural vegetation in protected areas throughout Paraguay provides desirable sites for sampling and understanding the eco-dynamics of hantaviruses spread in their rodent hosts. Additionally, an immense diversity of hantaviruses have been identified across the various biomes within Paraguay [1]. Herein, we conducted collections in western (Boquerón department) and southern (Itapúa department) Paraguay. Boquerón sits in the Dry Chaco (Figure 1B), a biodiverse region that has lost about twenty-seven percent of its original forest cover [44,45]. Itapúa sits in southern Paraguay (Figure 1B) and has a vegetation typical of the Interior Atlantic Forest of Brazil, northeastern Argentina, and eastern Paraguay.

Previously, we conducted prevalence and surveillance studies within the Reserva Natural de Bosque Mbaracayú (RNBM), Canindeyú department, a protected reserve of the Interior Atlantic Forest between 2014 and 2017 [34,46]. We found that hantavirus prevalence ranged from 4 to 12% [34] and was not influenced by species diversity, resource augmentation, or predator exclusion [34,46]. However, we found an association between significantly degraded habitats and prevalence levels in *Akodon montensis*, but not in *O. nigripes*. This may be explained by the generalist behavior of *O. nigripes* [34]. Consistently, we found that female rodents were less likely to be infected, with significantly higher odds of an adult male infection [34,47].

To understand the genetic relationship and spatiotemporal spread of JUQV in Paraguay, we used deep sequencing to screen and identify JUQV in *Oligoryzomys* sp. in two additional sites in Paraguay. Using the TN93 nucleotide substitution model, the coalescent (constant-size) population tree model, and Bayesian inference implemented in the Bayesian evolutionary analysis by sampling trees (BEAST) framework, we estimated the spatiotemporal dynamics of the JUQV strains we identified in eastern Paraguay.

## 2. Materials and Methods

### 2.1. Rodent Collection

Rodents were collected between February and March 2019 in southern Paraguay (Itapúa) and June 2019 in western (Boquerón) Paraguay. For the Itapúa department, five lines of 50 Sherman traps (7.6 × 8.9 × 22.9 cm, Sherman Trap Company, Tallahassee, FL, USA) and one line of approximately 34 traps were set for three to five nights in Hostettler property, Nueva Gambach; additionally, six lines of 50 traps were set for one to two nights in Ape Aimé (Figure 1B). For the Boquerón department, three sites were sampled: five lines for five nights and two lines for three nights in Rodeo Trebol; five lines for five nights and one line for four nights in Yalve Sanga; and, finally, three lines for five nights and two lines for four nights in Filadelfia (outskirts, east side, Figure 1B). All lines in the Boquerón department had 50 Sherman traps, totaling 23,400 trap nights. Collected rodents were euthanized, and their organs (including the kidney, heart, lungs, spleen, blood, urine, feces, and saliva) were snap-frozen in liquid nitrogen and stored at −80 °C until RNA extraction and sequencing. Mammal collection was conducted under permit 086/2019, and frozen tissues were exported under permits 049/2019 and 112/2019 from the Secretariat of the Environment (SEAM-Paraguay). Collection protocols followed the guidelines of the American Society of Mammalogists for the use of wild mammals in research and education [49].

### 2.2. Confirmation of Rodent Species

The confirmation of small mammal species (*Oligoryzomys* spp.) was performed by molecular methods and rodent morphologically, as described previously [34,47,50]. Briefly, approximately 10 mg sections of muscle or liver were used for DNA extraction with a MagMax Ultra DNA Extraction Kit 2.0 following the manufacturer’s protocol (Thermo Fisher Scientific, Waltham, MA, USA). DNA was quantified using the Qubit dsDNA High-Sensitivity Kit (Thermo Fisher Scientific), and 200 ng of DNA template was used for polymerase chain reaction (PCR). PCR was carried out using the forward (5′-GACAATCATACGAAAAAAYCACCCC-3′) and reverse (5′-AGTCTTCATTTTTGACTTACAAGGC-3′) primers for the cytochrome b gene under the following cycling conditions: an initial denaturation at 98 °C for 30 s; 35 cycles of denaturation at 98 °C for 10 s, annealing at 60 °C for 30 s, and extension at 72 °C for 30 s; and a final extension step at 72 °C for 5 min. PCR products were gel-purified and sequenced using Sanger sequencing by Eurofins Genomics.

### 2.3. Immunofluorescence Assay (IFA)

IgG antibodies to hantavirus in rat blood were detected using an immunofluorescence assay (IFA), as previously described [34,47]. Briefly, Vero E6 cells were infected with ANDV at an MOI of 0.1 and incubated at 37 °C and 5% CO_2_ for 7 days. The supernatant was removed and cells were trypsinized for 3 min and neutralized with an equal volume of growth media. Forty microliters of the cell suspension was deposited on each well of a 10-spot slide and incubated overnight at 37 °C and 5% CO_2_. Infected cells were fixed in acetone and stored at −80 °C until use. Nine 2-fold dilutions (1:32–1:4096) of blood samples from each rodent were prepared, and 20 µL of each dilution were deposited on their respective spots. One spot was used as a negative control for each slide. Slides were incubated for 30 min at 37 °C and washed three times in DPBS. The slides were incubated with 20 µL of a 1:500 dilution of the secondary antibody (Alexa Fluor^TM^ 488 F(ab^+^)2, an equal mixture of FITC-labeled anti-rat IgG and FITC anti-mouse IgG, (Life Technologies, Eugene, OR, USA) deposited on each spot for 30 min at 37 °C and washed three times in DPBS. Slides were observed under the microscope, and serology-positive samples were selected for next-generation sequencing.

### 2.4. Primer Design and Pooling

Based on our previous studies [51], degenerate primers targeting 0.5–1 kb amplicons of the JUQV reference from TK186690 (GenBank # S segment: OR184117, M segment: OR184122) were designed for initial amplification. Reads were mapped to the JUQV reference, and consensus sequences for each sample were obtained. The consensus sequences obtained were aligned, and primers were redesigned to target 0.5–1.0 kb amplicons of the nucleotide sequence of the S and M segments, resulting in 10 and 17 pairs of primers for the S and M segments, respectively (Table S1). Primers for the S and M segments were pooled into five groups for the forward and reverse primers, respectively: three containing seven to eight primers each and targeting 0.5 kb amplicons, and two containing two primers each and targeting 1.0 kb amplicons.

### 2.5. RNA Extraction and cDNA Synthesis

Total RNA was extracted from lung tissue using a MagMax Mirvana Total RNA Isolation Kit with the Kingfisher Flex System (Fisher Scientific) following the manufacturer’s protocol. Approximately 10 mg of frozen lung tissue was homogenized using the Bead Rupter 4 Homogenizer (Omni International, Kennesaw, GA, USA) at 5 m/s for 10 s, repeated three times (a total of 30 s). RNA was eluted in 50 µL of elution buffer and then heated at 65 °C for 5 min. RNA yield was quantified using a Qubit RNA Broad Range Assay kit (Thermo Fisher Scientific), and 500 ng input was used for cDNA synthesis with a Superscript IV First Strand Synthesis kit (Thermo Fisher Scientific). cDNA was synthesized using multiplexed forward primer pools for vRNA-specific amplification (Table S1). Primers were added to the reaction to reach a final concentration of 0.03 µM per primer. cDNA synthesis was performed in 20 µL reactions according to the manufacture’s protocol (Thermo Fisher Scientific).

### 2.6. Polymerase Chain Reaction (PCR)

Multiplex PCR was conducted in five reactions using the forward and reverse primer pools (Table S1) and 12.5 µL Platinum SuperFi PCR Master Mix (Thermo Fisher Scientific) in 25 µL (25 ng cDNA) volumes. The mixture was run in a PTC-200 Gradient Cycler (MJ Research) using the following cycling conditions: initial denaturation at 98 °C for 30 s; 30 cycles of denaturation at 98 °C for 10 s, annealing at 60 °C for 30 s, and extension at 72 °C for 15 s; and a final extension step at 72 °C for 5 min. PCR products from each reaction were combined and purified using a 0.8× ratio of AMPure XP beads (Beckman Coulter, Brea, CA, USA) per µL of PCR product. DNA yield was quantified using the Qubit dsDNA High-Sensitivity Assay kit (Thermo Fisher Scientific) and the Qubit^®^ Fluorometer (Thermo Fisher Scientific) and normalized to 1 ng/µL for each sample.

### 2.7. Library Preparation and Next-Generation Sequencing (NGS)

For each sample, five nanograms of DNA from the normalized PCR was used as input for DNA library preparation via a Nextera XT DNA Library Prep Kit (Illumina, San Diego, CA, USA). Briefly, PCR products were fragmented and tagged using 5 µL of amplicon tagmentation mix (ATM) for 5 min and then neutralized using 5 µL of NT buffer. Fragment ends were ligated with Set D indexes, and products were amplified using the following conditions: a heating step of 72 °C for 3 min; initial denaturation at 95 °C for 30 s; 12 cycles of denaturing at 95 °C for 10 s, annealing at 55 °C for 30 s, and extension at 72°C for 30 s; and a final extension at 72 °C for 5 min. PCR products were purified using AMPure XP beads (Beckman Coulter, Brea, CA, USA) while also double-sided size selected to obtain fragments with an average library size of around 500 bp as follows: right-sided size selection was performed at a 0.5× ratio by adding 22.5 µL of AMPure XP beads (Beckman Coulter) to 45 µL of the sample; left-sided size selection was performed by adding eight microliters of AMPure XP beads to 60 µL of supernatant (0.7× ratio) to bind smaller fragments. DNA yield was quantified using a Qubit dsDNA High-Sensitivity Assay kit (Thermo Fisher Scientific) and a Qubit^®^ Fluorometer (Thermo Fisher Scientific). The average library size was determined using the Agilent High-Sensitivity DNA kit or the Agilent 2100 Bioanalyzer System and then normalized to 4 nM for each sample. Each sample library was pooled at an equal volume, denatured, then pooled with a 5% PhiX (Illumina, San Diego, CA, USA) control. Sequencing was performed on the MiSeq (Illumina) using a MiSeq Reagent Kit v3 (150 cycles) (Illumina). FASTA files of paired-end reads that were generated from the Illumina MiSeq platform were analyzed using a CLC Genomics Workbench v22 (Qiagen, Hilden, Germany). Consensus sequences, with a minimum depth of 100 on a scale of 1000 and a Phred score of 30 were submitted to GenBank (OR184117–OR184126 and OR184959–OR184992).

### 2.8. Data and Sequence Analyses

To measure small mammal species diversity at each site, the Shannon diversity index (*H*) [52] was estimated using the vegan package [53] in R (v4.2.2). The Shannon equitability index (*E_H_*), was calculated for each line using the following equation:

*E_H_* = *H*/ln(*S*), (1)

where *E_H_* is the Shannon equitability index, *H* is the Shannon diversity index, and *S* is the number of species per sampling line.

### 2.9. Phylogenetic and Phylogeographic Analyses

The de novo assembly of JUQV S and M segments was performed using CLC genomic workbench v22 (Qiagen). Briefly, reads obtained from each of the serology-positive samples after primer redesign were subjected to de novo assembly in CLC genomic workbench v22. Full-length S and M sequences were obtained from TK186690, which was used as the reference sequence (GenBank # S segment: OR184117, M segment: OR184122) for mapping. JUQV sequences were aligned in MEGA software (v11.0.10) [54] using the MUSCLE program [55]. Nucleotide regions 20–1683 bp and 271–3451 bp of the S and M segments, respectively, containing the open reading frames (ORFs) were used for phylogenetic and phylogeographic [56,57] analyses in BEAST (v2.7.4) [58]. We employed the Bayesian Markov chain Monte Carlo (MCMC) process for our analyses, and our priors were largely non-informative. Through a posterior support obtained by averaging over all and eliminating uncertainties from different substitution models using bModelTest [59] software v1.3.3, we determined that TN93 was the best substitution-site model for our analyses (Figures S1 and S2). We used a strict molecular clock model and a coalescent (constant-size) population model [60], since Ramsden et al. showed no significant difference in clock rate estimates between strict and relaxed clocks [61], and the seroprevalence from our previous studies remained in a constant range (4–12%) [34,47]. The mutation rate was estimated by specifying the parameter in the “Site model” panel of BEAUti using the 1/X distribution and a fixed mean substitution rate of 1. The MCMC process was run for several iterations, in increasing order of chain length; then, the chain length (10^8^ generations) that had sufficient mixing and an effective sample size (ESS) after posterior distribution summary analysis using TRACER (v1.7.2) [62] was applied. The MCMC chain was sampled every 100,000 generations. The tree distribution summary was analyzed using DensiTree (v2.0.0) [63] after a 10% burn-in. A maximum clade credibility tree with a posterior probability (PP) limit of 1 was obtained using Tree Annotator (v2.7.3) and visualized using FigTree (v1.4.4) (http://tree.bio.ed.ac.uk/software/figtree/ accessed on 9 February 2023). Owing to the availability of geographical coordinate data for the samples obtained in our collection, we used a continuous phylogeographic model [57], as these are more appropriate for land-dwelling viral hosts (https://www.beast2.org/2022/03/01/phylogeography.html accessed on 30 January 2023). We also assumed a random walk on a sphere implemented in the GEO_SPHERE package in BEAST2. The phylogeographical reconstruction was visualized using SPREAD4 (https://spreadviz.org/home accessed on 30 March 2023) [64], and the spatiotemporal information embedded in the trees was obtained using the R package “Seraphim” [65] in R v4.2.3.

The partial coding sequence (CDS) of the S segment (270–1329 nucleotide (nt) regions) of all sequences in this study was aligned to thirty-three Central and South American hantavirus sequences published on GenBank (Table S2). Sequences from Sin Nombre virus (SNV) were included as an outgroup. Alignments were performed using MUSCLE with the default parameters in MEGAX [55,66]. ModelFinder [67] implemented in IQTREE [68] was used to select the best nucleotide substitution model to estimate evolutionary distances. The best candidate model was chosen based on Akaike’s information criterion (AIC). Phylogenetic trees were constructed using IQTREE on the Cipres Science Gateway server (http://www.phylo.org/ accessed on 17 August 2023) via the general time-reversible model with empirical base frequencies, invariable sites (0.5149), and a discrete Gamma model (1.134). The GTR + F + I + G model was run with 1000 ultrafast bootstraps [69]. Trees were visualized and edited in iTol v.6 [70].

## 3. Results

### 3.1. Rodent Sampling

A total of 612 small mammals (rodents and marsupials) were captured along linear trap lines; 358 from Boquerón (Table 1) and 254 from Itapúa (Table 2). A total of 25 native species were captured, with 14 species from Boquerón and 12 species from Itapúa. Species richness varied among the lines of collection, ranging from two in line G to nine in line E in Boquerón, and six in lines A and F to eight in lines B and D in Itapúa.

Diversity varied significantly among lines in Boquerón (µ = 1.467, df = 6, *p* < 0.0001) and Itapúa (µ = 1.43, df = 5, *p* < 0.0001) (Table 3). Species evenness was high in all lines but significantly different among lines in Boquerón (µ = 0.784, df = 6, *p* < 0.0001) and Itapúa (µ = 0.735, df = 5, *p* < 0.0001) (Table 3). The high evenness in line G of Boquerón was due to poor species richness (2), as the number of each species collected in line G did not differ significantly. Overall species diversity was higher in Boquerón than Itapúa (Table 3), and species evenness in both locations did not differ significantly. Our collection featured a high number of *Akodon toba* (33.5%)*, N. lasiurus* (26.8%), *Calomys laucha* (12.0%), and *Oligoryzomys chacoensis* (10.1%) in Boquerón and *Akodon montensis* (37.6%), *Akodon azarae* (26.0%)*,* and *O. nigripes* (19.3%) in Itapúa.

**Figure 2:**
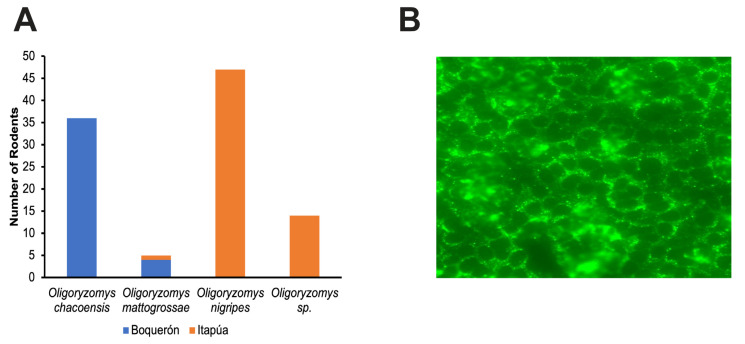
Summary of *Oligoryzomys* spp. collected from Boquerón and Itapúa and representative immunofluorescent image. (**A**) The total number of *Oligoryzomys spp* rodents captured from Boquerón (blue) and Itapúa (orange). (**B**) Epifluorescence microscopic image (20X) of a serologically positive rodent blood sample from one well of a 10-well spot slide. The spot slides were incubated with blood from each rodent, washed, and probed with Alexa Fluor^TM^ 488 F(ab^+^)2. Slides were observed using a Zeiss epifluorescence microscope, and cells with characteristic fluorescent punctate staining near the perinuclear region in at least 70% of the spot were deemed positive.

Of the 612 small mammals captured, 102 *Oligoryzomys* spp. were captured, with 60 from Itapúa and 42 from Boquerón (Figure 2A).

### 3.2. Confirmation of Infection by IFA and NGS

For IFA, slide spots with more than 70% of the cells presenting the characteristic punctate staining around the nucleus were deemed to be positive (Figure 2B). Of the 102 blood samples screened, three (3) samples were positive, with the lowest dilution being 1:128 (Table S3). All serology-positive samples were from the Itapúa department.

We obtained five RNA-positive rodent lung samples using next-generation sequencing. These five samples included three serology-positive samples and two RNA-positive/IFA-negative samples. All RNA-positive samples were from *O. nigripes* in the Itapúa Department.

Two full-length, high-quality sequences, with a minimum depth of 100 for the S and M segment sequences (Table S4), were used for alignments in MEGA software. Nucleotide regions 20–1683 bp and 271–3451 bp of the S and M segments, respectively, were used for phylogenetic analysis.

### 3.3. Phylogenetic and Phylogeographical Analyses

In addition to the two sequences (TK186690 and TK206269; Table S5) obtained from Itapúa, seventeen consensus sequences from our previous field studies in the department of Canindeyú in Paraguay (Table S5) were included in the phylodynamic analyses. All nucleotide sequences were obtained from lung samples of *O. nigripes,* except for the JUQV TK66745 strain, which was identified in *O. mattogrossae*. Together, we had a total of nineteen S and M segment sequences. We excluded M-segment sequences that had no S segment or an incomplete S segment, and vice versa for M-segment analysis. This was to ensure that each rodent reservoir was accounted for in the statistical output for all gene topologies in the analyses of both segments, hence also allowing comparability.

**Figure 3:**
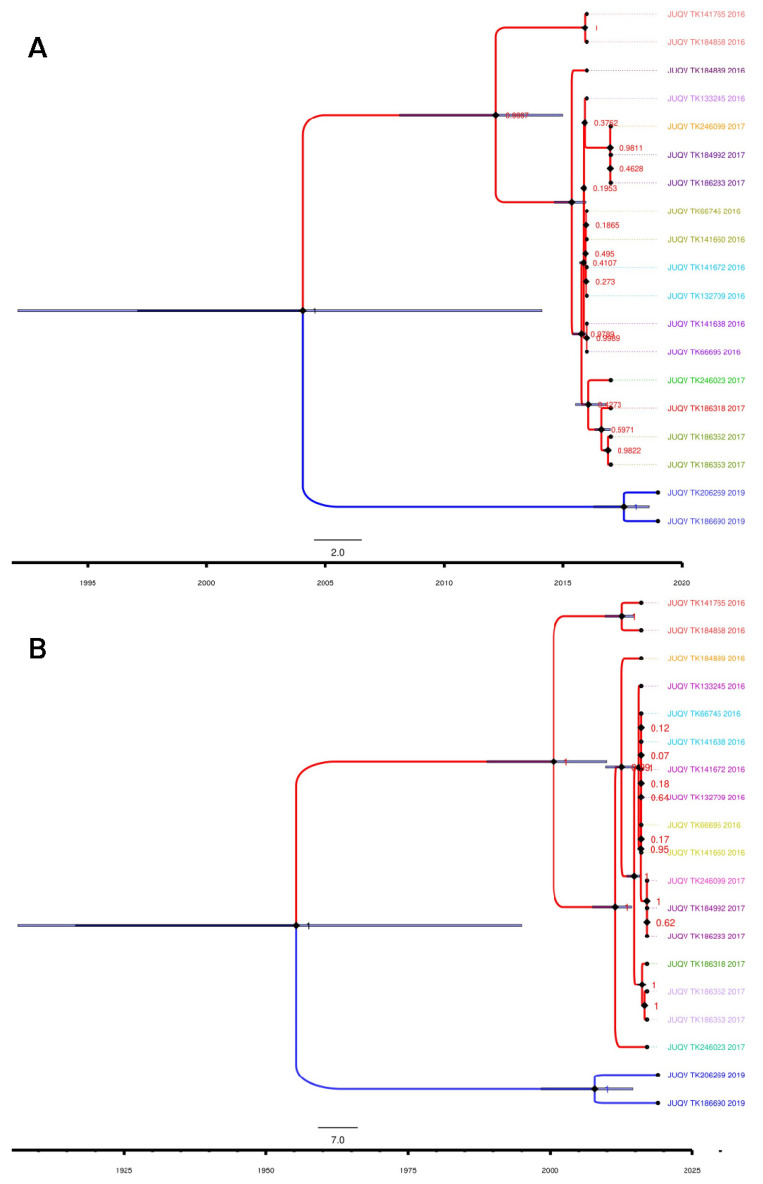
Strict molecular clock and maximum clade credibility trees for JUQV S and M segments. Illustrations were generated using BEAST output and annotated with the FigTree annotator. Phylogenetic relationships of JUQV (**A**) S segments and (**B**) M segments from Paraguay. Nodes are represented by dark diamond shapes and tips by dark circles. Blue bars across nodes represent 95% height posterior density. Blue and red taxa represent Itapúa and Canindeyú taxa, respectively. Tip labels with the same color indicate the same clade, and a colored tip without a pair indicates a higher clade. GenBank accession numbers for samples are provided in Table S4.

Our phylogenetic analysis revealed that the JUQV strains identified in Paraguay formed distinct monophyletic clades (Figure 3) based on location. The JUQV strains in Canindeyú formed a clade distinct from those in Itapúa (posterior probability (PP) = 1). This was true for both S (Figure 3A) and M (Figure 3B) segments, and most of the nodes in both segments. The JUQV strains in Canindeyú appeared to have diverged earlier than the strains in Itapúa, but this could have been due to the lower number of JUQV strains (n = 2) identified in Itapúa. The substitution rate for the S segment was determined to be 4.46 × 10^−4^ subs./site/year (95% height posterior density (HPD) = ~0.03–21.83). This was higher than that of the M segment, which was 7.88 × 10^−5^ subs./site/year (95% HPD = ~0.08–21.60), though both had sufficiently low variances (V) from their means (V_M-Segment_ = 1.166 × 10^−9^; V_S-Segment_ = 7.775 × 10^−8^). We estimated that the effective population size N*_e_* of the M segment (11.82, 95% HPD ~3.96–23.81) was larger than that of the S segment (2.01, 95% HPD ~0.37–4.36), and our estimates showed a mutation rate of 3.042 × 10^−3^ mut./year (95% HPD = 3.00 × 10^−5^–1.24 × 10^−2^ mut./year) for the S segment, which was less than that of the M segment at 9.028 × 10^−4^ mut./year (95% HPD = 8.58 × 10^−6^–3.18 × 10^−3^ mut./year). The time of the most recent ancestor (TRCA) of the S segment’s sequences was estimated to be 2004 (95% HPD = 1990–2014), and this differed significantly from that of the M segment, which was estimated to be 1954 (95% HPD = 1921–1999). The maximum likelihood (ML) phylogenetic analysis of the CDSs of the nucleocapsid of 52 New World hantaviruses showed that the JUQV identified in Paraguay clustered together in the tree, with very good node support (bootstrap values of 100) (Figure S3). The JUQV identified in Canindeyú formed a clade with human isolates of ARAV and JUQV. The JUQV identified in Itapúa also clustered with Itapúa viruses 37 and 38, and JUQV TK186690 appeared to be older than all the viruses from Itapúa in that clade. Overall, our ML phylogenetic analysis suggested that the evolution of JUQV occurred in a geographical context given the clustering of Paraguayan JUQV and hantaviruses identified in southern Brazil, which is proximal to our sites of sampling.

**Figure 4:**
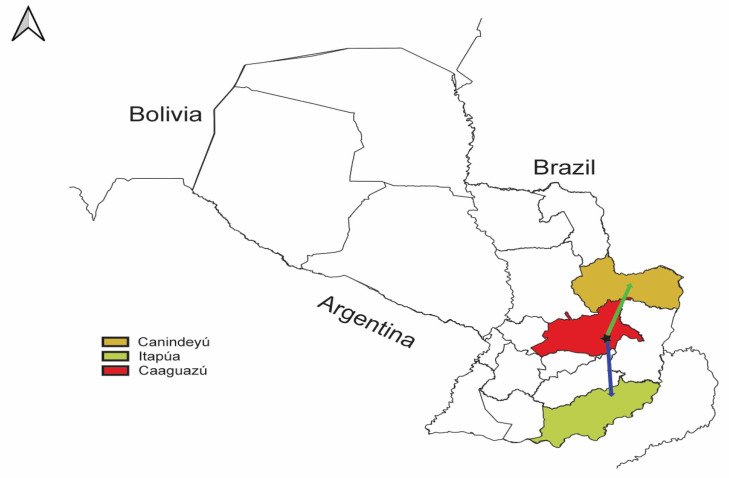
QGIS map showing phylogeography of JUQV in Paraguay. The illustration of the JUQV distribution was based on BEAST output visualized using SPREAD4. The black star represents the estimated location of the predicted most recent ancestor of JUQV in the Caaguazú department (red) in Paraguay. The arrows indicate the predicted migration to Itapúa (blue) and Canindeyú (yellow).

Our phylogeographic analysis predicted that the most recent ancestors of the S and M segments had a similar geographic origin (Figure 4, Figures S4 and S5), and they seemed to be confined in the Interior Atlantic Forest, which is the JUQV rodent reservoir’s primary biome. We obtained a median value for the weighted branch dispersal velocity of ~72.35 km (95% HPD ~20.34–194.95 km/year) (Figure S6A) for the S segment and ~13.32 km/year (95% HPD ~4.28–30.45 km/year) (Figure S6B) for the M segment. The median value for the weighted diffusion coefficient was 1871.32 km/year (95% HPD ~280.70–13,723.72 km/year) (Figure S6C) for the S segment and 254.82 km/year (95% HPD ~34.01–1607.12 km/year) (Figure S6D) for the M segment. This is an ecological measure of the intrinsic diffusivity of infected individuals that reflects the area an infected host will explore per unit time, initially defined by Pybus et al. [71] and later modified by Trovao et al. [72] to obtain lower estimates and less variance. Also, we estimated a spatial distance traveled of ~200 km (95% HPD ~150–400 km) (Figure S7A) and a patristic distance of ~500 km (95% HPD ~300–1500 km) (Figure S7B) from the epidemic origin for the S segment, and a spatial distance traveled of ~200 km (95% HPD ~150–300 km) (Figure S7C) and a patristic distance of ~450 km (95% HPD ~300–750 km) (Figure S7D) from the epidemic origin for the M segment.

## 4. Discussion

Since the first isolation of HTNV [3], enormous efforts have been channeled towards understanding hantavirus’ evolution [73,74,75,76]. The most common school of thought has been that hantaviruses have co-speciated with their rodent hosts [77]. This theory rests on the fine congruence formed between the rodent reservoirs’ cytochrome B gene and hantavirus’ genome sequences in phylogenetic analysis [77]. In recent years, more sampling has led to the identification and characterization of hantaviruses in other hosts, such as shrews [4,78,79,80,81] and bats [82,83,84,85]. The phylogenetic analysis of these hantaviruses has shown that hantaviruses identified in shrews and bats comprise the basal part of phylogenetic trees [73,83] and exhibit more diversity compared to hantaviruses identified in rodent hosts, suggesting more complexity [75] and perhaps indicating that these are more ancient hosts of hantaviruses [74]. Some rodent borne hantaviruses have been identified in more than one rodent reservoir, suggesting their ability to jump to other rodent species, although whether the virus persists in these other rodents remains unknown [76]. JUQV-like viruses have been identified in *Akodon cursor*, *A. montensis, A. paranaensis*, *O. mattogrossae*, *Oxymycterus judex*, and *Thaptomys nigrita* [35,86,87,88]. Over the past 50 years, much hantavirus research has been biased towards those with rodent reservoirs [89], and thus our understanding of the history of hantavirus evolution is still poor in the context of all mammals that harbor these viruses. One problem for all reservoirs, including rodents, is the limited number of complete genomes [77], and hence we need to greatly expand the identification and characterization of hantaviruses in all possible hosts. This study provided insight into the S and M segments of JUQV in *O. nigripes* in different regions of Paraguay, and future efforts will aim to obtain the L segment as well as to expand our knowledge regarding its spatiotemporal evolutionary history.

Following the outbreak of HCPS in Paraguay caused by LANV in 1995 [16], we have continued the surveillance, identification, and characterization of hantaviruses in sympatric reservoirs in Paraguay [12,32,90]. However, the evolution and ecological dynamics of hantaviruses may be specific to each hantavirus. We have seen this specificity with the modes employed by different hantaviruses for entry [91,92] and the ability of certain hantaviruses to encode non-structural proteins [93]. Studies exploring the migration patterns of rodent reservoirs with their specific hantaviruses are greatly needed, since the diversification of each hantavirus is largely a result of the migration patterns of viruses and their hosts [94]. Our study employed a continuous phylogeographical model because of issues such as the unrealistic subdivision of sampling sites, the effect of ancestor restriction to sampled sites on the inferred dispersal history, and inadequate estimates due to over- or under-sampling seen with discrete models, although the latter can also affect continuous models [57,95,96,97,98]. We also employed a coalescent (constant-size) population model because our data suggested that hantavirus seroprevalence at our study sites mostly ranged between 4 and 12% over time [34,47]. Aside from these assumptions, our priors were all non-informative. This represented a “bottom-up” approach to understanding hantavirus evolution as compared to the use of informative priors, which could bias inference related to the spatiotemporal dynamics of specific hantaviruses. Some studies, however, have explored the spatiotemporal dynamics of a group of hantaviruses using a discrete phylogeographic model [99], as well as a unique hantavirus, Tula virus (*Orthohantavirus tulaense*, TULV), employing a continuous phylogeographic model [94].

*Oligoryzomys nigripes* is the primary reservoir of JUQV and is a habitat generalist distributed in the Cerrado and Atlantic Forest biomes of Argentina, Uruguay, Brazil, and Paraguay [89]. The phylogenetic analysis of complete S-segment sequences of JUQV identified in O. nigripes revealed two distinct lineages of JUQV in Brazil: an endemic and non-endemic clade [100]. The non-endemic clade had high sequence homology and was identified in Espírito Santo and Rio de Janeiro, Brazil. The endemic clade had widespread geographic distribution, spanning areas in Argentina, Brazil, and Paraguay, south of the non-endemic regions [100]. The analysis showed that JUQV isolated from Paraguay formed a clade with JUQV strains isolated from human HCPS cases in Parana, Brazil. Another study identified a possible third lineage in Brazil and the co-circulation of JUQV and ARAV, though the consequence of this co-circulation on viral diversity is not yet understood [26,33]. Our study took advantage of the availability of geographic sampling coordinates and explored the spatial and temporal history of JUQV identified in Paraguay. We estimated the time to the most recent ancestor (TMRA) for the S and M segments, as well as the possible location of the ancestor/founder JUQV.

The previous phylogenetic analysis of 190 S-segment sequences (1319 bp) from diverse species and locations estimated the substitution rate to be 6.8 × 10^−4^ subst./site/ year [99]. Although our analysis used a different substitution model, we estimated substitution rates that were in the same range as this estimation, with small variances for JUQV. Some details of note are that: (a) the previous phylogenetic analysis of the S segment from diverse regions used an informative time scale based on a previously determined substitution rate of 10^−2^ to 10^−4^ [61]; (b) the estimate was collective rather than specific [99]; and (c) the sequences for Sigmodontine rodent-borne hantaviruses used from the source that informed their prior were partial-length sequences [61]. Notwithstanding, our estimate was in the range observed by Ramsden et al., who estimated a substitution rate concordant with other RNA viruses at 10^−2^ to 10^−4^ subs/site/year [61]. Our effective population size estimates for the S segment were approximately five-fold lower than those of the M segment, even though the substitution rate for the S segment was higher. Considering that the M segment had an older ancestor compared to the S segment, it was not surprising to have a population size higher than that of the S segment. It is of interest that the S segment had a higher substitution rate, even though its substitution rate was typical of the predicted rate for hantaviruses and RNA viruses. Studies comparing the evolutionary rates of hantavirus genomic segments are scarce; nevertheless, a study investigating the evolutionary rates of 751 and 142 nucleotide regions of the S and M segments of SNV, respectively, suggested close absolute substitution rates (1.93 × 10^−3^ and 6.76 × 10^−3^ for the S and M segments, respectively). However, the authors found that the replacement substitution rates in the S segment (1.73 × 10^−3^) and M segment (7.0 × 10^−4^) were higher compared to the average rates of synonymous substitutions (0.312 and 0.378 for the S and M segments, respectively), suggesting strong purifying selection. The replacement substitution rate was, however, higher in the S segment compared to the M segment [101]. Another study explored the molecular evolution of Seoul virus (*Orthohantavirus seoulense*, SEOV) using partial (429–1331 nt region) and complete (36–3447 nt region) CDS [102]. The authors estimated similar substitution rates for the S and M segments of SEOV identified in rats in an urban city in China, but with slightly higher rates in the S segment (4.2126 × 10^−4^) than in the M segment (2.0631 × 10^−4^). The 95% HPD values suggested that the substitution rates in the M segment (9.9816 × 10^−5^–4.0076 × 10^−4^) could reach as low as 10^−5^, but not in the S segment (1.8639 × 10^−4^–5.9634 × 10^−4^) [102]. Despite this, there was more variability in the M segment on a nucleotide level. The mutation rate in both segments (Mut._M-segment_ = 9.028 × 10^−4^, Mut._S-segment_ = 3.042 × 10^−3^) followed the same pattern as the substitution rate, although the ESS for the M segment in both cases was not appreciably sufficient. Moreover, since the substitution was higher in the S segment and the resulting mutation and variability were not seen in the JUQV S segment, as with the M segment, there must be a negative selection for certain nucleocapsid variants of JUQV carried by infected rodents in Paraguay. This highlights the need to query each genomic segment and mutation that drives selection in the JUQV population. Also, the N*_e_* is always underestimated, since the coalescent model does not take population dynamics into account [103]. However, it should be noted that the N*_e_* in viral phylodynamics describes the effective number of infections—a quantity proportional to the number of infected individuals, which gives the same coalescent rate (driven primarily by incidence) as for an ideal population [104]. The phylogenetic relationships of the JUQV sequences from the lungs reported herein were compared with other JUQV S-segment sequences from Brazil (Figure S3). The JUQV identified in Canindeyú formed a clade with JUQV lineages from HCPS-endemic regions in southeastern Brazil. A clade-defining mutation was identified at residue 382. Clade 1 sequences had a leucine (L) at position 382, while Clade 2 sequences had a methionine (M) at this position. This was consistent with previous findings examining the phylogenetic relationship of the S segments of JUQV identified in *O. nigripes* [100]. Studies examining the phenotypic consequences of these genotypes in JUQV infection are necessary. The 2019 sequences from Itapúa clustered with the previous sequences isolated in 1998 from Itapúa. However, the JUQV identified in 2019 was more basal in the tree and hence older. This further highlights the need for more sampling, which would not only recover more JUQV circulating in these biomes but calibrate the molecular clock in future analyses.

Our spatiotemporal analysis showed that although the temporal dynamics of divergence times, genetic events, and population kinetics differed in both segments of the virus, they shared a common spatial history and origin. We estimated that the most recent ancestor would be located in the Caaguazú department (Figure 4), a department that was not included in our sampling sites but is located in the Interior Atlantic Forest ecoregion of Paraguay. Longitudinal studies evaluating *O. nigripes* migration patterns are scarce. However, it has been estimated that the mean maximum distance moved (MMDM) by *O. nigripes* is twenty-two meters/day [105]. Although most field studies do not capture movements beyond the study site, *O. nigripes* movement can range from 18.04 to 31.81 m/day for females and males, respectively [106], with a higher frequency (60%) of short-distance movement (0–20 m) than long-distance movement (15%, 20–80 m) [107]. Since males are the major drivers of infection [34,47], this would sum to approximately eleven kilometers/year. Hence, the transmission of infection and genetic events such as divergence observed in our phylogenetic analysis must involve reservoir hosts not captured in our sampling, considering the estimated branch dispersal velocity in both segments. Moreso, it must require more than what we captured in our sampling to cover the distances estimated for the epidemic wavefronts (spatial and patristic distances) for the S and M segments. This was further highlighted by the diffusion coefficient estimated for the S and M segments, although we do not know the average distance traveled in the lifetime of *O. nigripes*.

We explicitly examined JUQV’s spatiotemporal dynamics outside the context of its host. This approach has been suggested previously as a necessary step to establish the co-speciation of hantaviruses with their rodent hosts [61], and the importance of evaluating each hantavirus species differently has been demonstrated previously [108]. It has also been shown that an increased number of JUQV genome sequences would increase the clarification of JUQV evolution [108], and although there are statistical methods for increasing the temporal spread of sampling times, hence increasing statistical power for coalescent methods [77,109,110], it is clear that more sampling of these viruses in their hosts would inform the best evolutionary model. Also important would be the sampling of hantaviruses from host reservoir fossils; this would not only increase our breadth of knowledge on the history of hantaviruses, but also aid in calibrating molecular clocks for the analysis of JUQV genome sequences isolated between short time intervals [109]. The calibration of the time scale using rodent fossils would highlight the gap in events that occurred between our current data and the founder JUQV strain [77,110]. This is because, given the high rates of RNA virus mutations, sampling over short periods would only capture a few significant mutations [61].

## 5. Conclusions

Given the fast mutation rates of the S and M segments of the JUQV identified and the estimated age of the most recent ancestors, it is evident that there were evolutionary events not represented in our spatiotemporal analyses. Our study highlighted that more data are needed to understand and describe the temporal and spatial evolutionary events that have occurred within the Paraguayan JUQV population. To address this gap, we propose the sampling and sequencing of entire genomes from hantaviruses throughout the Interior Atlantic Forest of Paraguay and beyond. With more sequence data and statistical tools such as Bayesian inference, data will become available to dissect the hantavirus evolutionary mystery.

## Figures and Tables

**Table 1:** Small mammal species collected per line in Boquerón, Paraguay (June 2019).

Lines	A	B	C	D	E	F	G	Total
Species	(N = 66)	(N = 68)	(N = 68)	(N = 50)	(N = 67)	(N = 36)	(N = 3)	(N = 358)
**Family:** Cricetidae								
*Akodon toba*	23 (34.8%)	22 (32.4%)	30 (44.1%)	11 (22.0%)	32 (47.8%)	2 (5.6%)	0 (0%)	120 (33.5%)
*Andalgalomys pearsoni*	0 (0%)	1 (1.5%)	0 (0%)	0 (0%)	0 (0%)	0 (0%)	0 (0%)	1 (0.3%)
*Calomys callosus*	2 (3.0%)	5 (7.4%)	2 (2.9%)	0 (0%)	3 (4.5%)	0 (0%)	0 (0%)	12 (3.4%)
*Calomys laucha*	5 (7.6%)	14 (20.6%)	11 (16.2%)	7 (14.0%)	2 (3.0%)	2 (5.6%)	2 (66.7%)	43 (12.0%)
*Graomys chacoensis*	8 (12.1%)	3 (4.4%)	2 (2.9%)	4 (8.0%)	6 (9.0%)	2 (5.6%)	0 (0%)	25 (7.0%)
*Holochilus chacarius*	0 (0%)	0 (0%)	1 (1.5%)	0 (0%)	0 (0%)	6 (16.7%)	0 (0%)	7 (2.0%)
*Necromys lasiurus*	15 (22.7%)	12 (17.6%)	15 (22.1%)	21 (42.0%)	11 (16.4%)	21 (58.3%)	1 (33.3%)	96 (26.8%)
*Pseudoryzomys simplex*	0 (0%)	0 (0%)	0 (0%)	1 (2.0%)	0 (0%)	0 (0%)	0 (0%)	1 (0.3%)
*Oligoryzomys chacoensis*	11 (16.7%)	5 (7.4%)	5 (7.4%)	3 (6.0%)	10 (14.9%)	2 (5.6%)	0 (0%)	36 (10.1%)
*Oligoryzomys mattogrossae*	0 (0%)	3 (4.4%)	0 (0%)	0 (0%)	0 (0%)	1 (2.8%)	0 (0%)	4 (1.1%)
**Family:** Didelphidae								
*Gracilinanus agilis*	1 (1.5%)	0 (0%)	0 (0%)	0 (0%)	0 (0%)	0 (0%)	0 (0%)	1 (0.3%)
*Monodelphis kunsi*	1 (1.5%)	0 (0%)	0 (0%)	0 (0%)	0 (0%)	0 (0%)	0 (0%)	1 (0.3%)
*Thylamys pusillus*	0 (0%)	2 (2.9%)	2 (2.9%)	3 (6.0%)	2 (3.0%)	0 (0%)	0 (0%)	9 (2.5%)
**Family:** Caviidae								
*Galea musteloides*	0 (0%)	1 (1.5%)	0 (0%)	0 (0%)	1 (1.5%)	0 (0%)	0 (0%)	2 (0.6%)

The number before the parentheses represents the total number of species collected in that line. In parenthesis is the proportion of each species in that line (represented as a percentage). Percentages are rounded to their nearest significant number.

**Table 2:** Small mammal species collected per line in Itapúa, Paraguay (February-March 2019).

Lines	A	B	C	D	E	F	Total
Species	(N = 37)	(N = 39)	(N = 50)	(N = 41)	(N = 31)	(N = 56)	(N = 254)
**Family:** Cricetidae							
*Akodon azarae*	12 (32.4%)	21 (53.8%)	12 (24.0%)	4 (9.8%)	7 (22.6%)	10 (17.9%)	66 (26.0%)
*Akodon montensis*	16 (43.2%)	5 (12.8%)	22 (44.0%)	18 (43.9%)	5 (16.1%)	30 (53.6%)	96 (37.8%)
*Akodon* sp.	0 (0%)	0 (0%)	0 (0%)	1 (2.4%)	0 (0%)	0 (0%)	1 (0.4%)
*Euryoryzomys* sp.	0 (0%)	0 (0%)	1 (2.0%)	0 (0%)	0 (0%)	0 (0%)	1 (0.4%)
*Mus musculus*	1 (2.7%)	2 (5.1%)	0 (0%)	1 (2.4%)	9 (29.0%)	1 (1.8%)	14 (5.5%)
*Oligoryzomys flavescens*	1 (2.7%)	2 (5.1%)	0 (0%)	2 (4.9%)	0 (0%)	2 (3.6%)	7 (2.8%)
*Oligoryzomys mattogrossae*	0 (0%)	2 (5.1%)	1 (2.0%)	2 (4.9%)	1 (3.2%)	0 (0%)	6 (2.4%)
*Oligoryzomys nigripes*	6 (16.2%)	5 (12.8%)	12 (24.0%)	7 (17.1%)	9 (29.0%)	10 (17.9%)	49 (19.3%)
*Oligoryzomys*	0 (0%)	1 (2.6%)	0 (0%)	0 (0%)	0 (0%)	0 (0%)	1 (0.4%)
*Sooretamys angouya*	0 (0%)	1 (2.6%)	1 (2.0%)	0 (0%)	0 (0%)	0 (0%)	2 (0.8%)
*Thaptomys nigrita*	0 (0%)	0 (0%)	0 (0%)	6 (14.6%)	0 (0%)	3 (5.4%)	9 (3.5%)
**Family:** Didelphidae							
*Monodelphis dimidiata*	1 (2.7%)	0 (0%)	1 (2.0%)	0 (0%)	0 (0%)	0 (0%)	2 (0.8%)

The number before the parentheses represents the total number of species collected in that line. In parenthesis is the proportion of each species in that line (represented as a percentage). Percentages are rounded to their nearest significant number.

**Table 3:** Estimates of rodent species diversity indicators in Boquerón and Itapúa.

Location	Parameter	A	B	C	D	E	F	G	Overall
**Boquerón**	Shannon diversity (*H*)	1.69	1.88	1.55	1.59	1.56	1.35	0.63	1.82
	Shannon equitability (*E_H_*)	0.81	0.79	0.75	0.82	0.71	0.70	0.91	0.69
**Itapúa**	Shannon diversity (*H*)	1.32	1.50	1.36	1.65	1.50	1.30	N/A	1.64
	Shannon equitability (*E_H_*)	0.73	0.72	0.70	0.79	0.75	0.72	N/A	0.66

N/A: “not applicable”.

## Data Availability

All data are available in the main text or Supplementary Materials.

## Supplementary Materials

The following supporting information can be downloaded at: https://www.mdpi.com/article/10.3390/v15091798/s1, Figure S1: S-segment support for substitution model used, Figure S2: M-segment support for substitution model used, Figure S3: New World nucleocapsid (S) maximum likelihood tree, Figure S4: SpreadViz 4 spatial spread visualization for M segment, Figure S5: SpreadViz 4 spatial spread visualization for S segment, Figure S6: Summary of estimated dispersal statistics, Figure S7: Summary of estimated dispersal statistics (epidemic wavefronts), Table S1: Forward (Pool-F) and reverse (Pool-R) primer sequences and pool designations for JUQV nucleotide sequences, Table S2: S-segment hantavirus reference sequences, Table S3: Reciprocal IFA titers, Table S4: Summary of reads, sequencing depths, and coverage for the S and M segments from selected lung samples, Table S5: Sample numbers and GenBank accession numbers used for phylogenetic and phylogeographic analyses of S and M segments.
