# Supplementary material for: Deep Sequencing to Reveal Phylo-Geographic Relationships of Juquitiba Virus in Paraguay
Source: Viruses. 2023 Aug 24;15(9):1798. doi: 10.3390/v15091798 (PMC10537311; doi:10.3390/v15091798)
Supplement: Supplementary file 1 [file viruses-15-01798-s001.zip › Supplementary Figures.pdf]

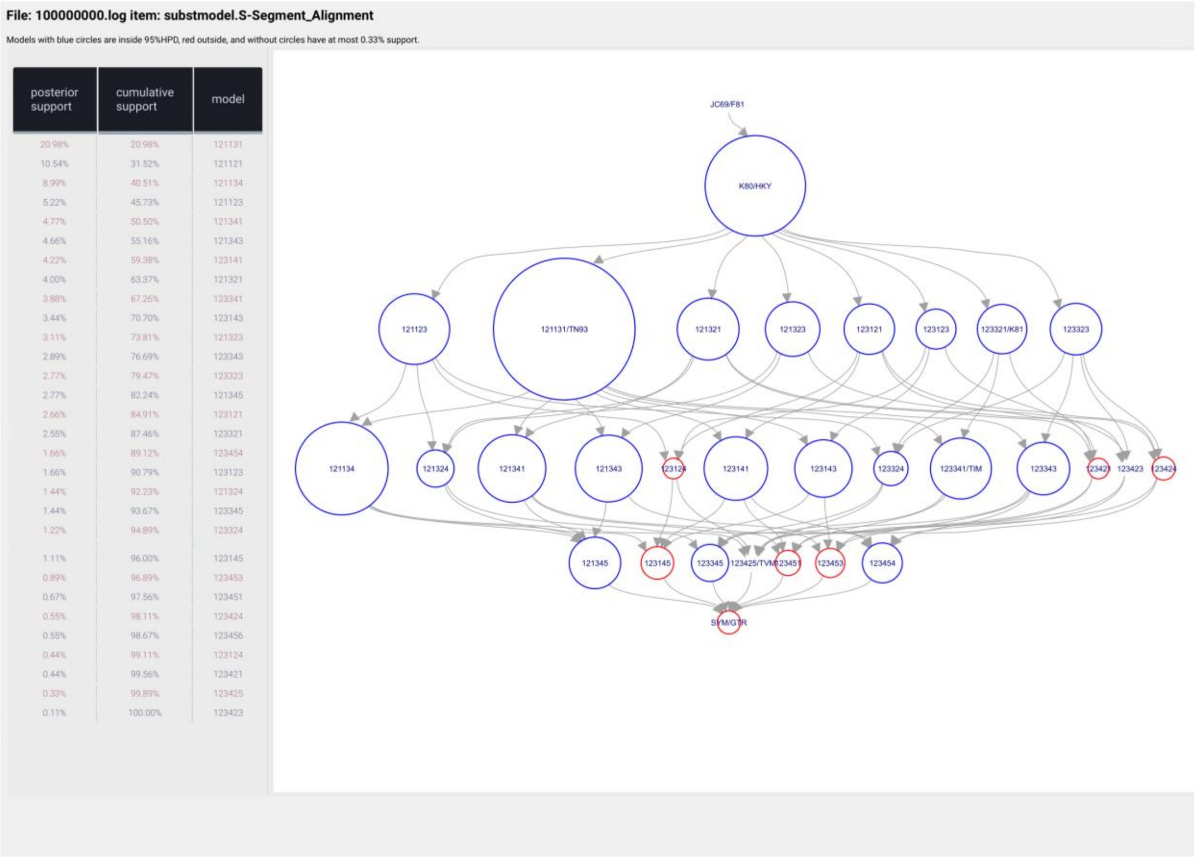

posterior support	cumulative support	model
20.98%	20.98%	121131
10.54%	31.52%	121121
8.99%	40.51%	121134
5.22%	45.73%	121123
4.77%	50.50%	121341
4.66%	55.16%	121343
4.22%	59.38%	123141
4.00%	63.37%	121321
3.88%	67.26%	123341
3.44%	70.70%	123143
3.11%	73.81%	121323
2.89%	76.69%	123343
2.77%	79.47%	123323
2.77%	82.24%	121345
2.66%	84.91%	123121
2.55%	87.46%	123321
1.66%	89.12%	123454
1.66%	90.79%	123123
1.44%	92.23%	121324
1.44%	93.67%	123345
1.22%	94.89%	123324
1.11%	96.00%	123145
0.89%	96.89%	123453
0.67%	97.56%	123451
0.55%	98.11%	123424
0.55%	98.67%	123456
0.44%	99.11%	123124
0.44%	99.56%	123421
0.33%	99.89%	123425
0.11%	100.00%	123423

Figure S1. S segment support for substitution model used. Model support was obtained by uploading the log file from BEAST 2.7.4 output into the BModelAnalyzer program in the AppLauncher software of BEAST 2.7.4. Each circle represents a substitution model, and the size of each circle represents the degree of support.

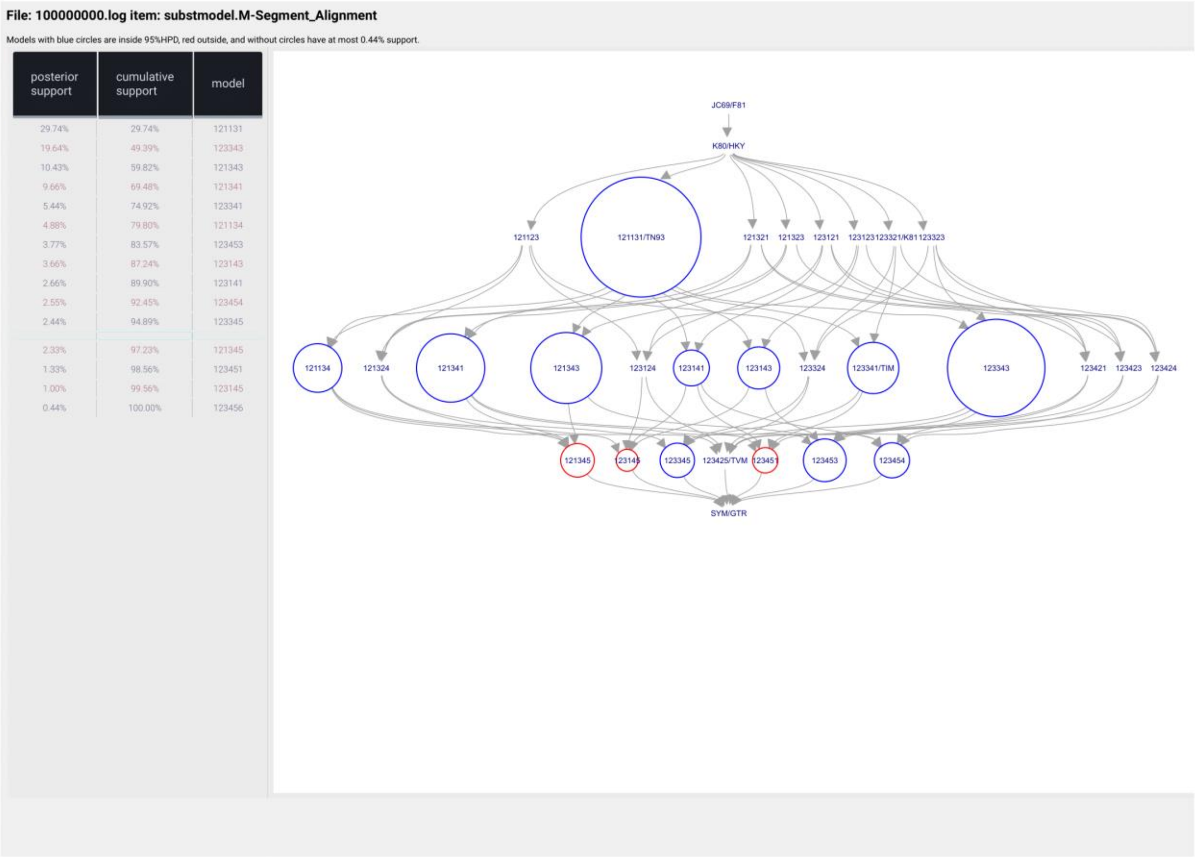

Figure S2. M segment support for substitution model used. Model Support was obtained by uploading the log file from BEAST 2.7.4 output into the BModelAnalyzer program in the AppLauncher software of BEAST 2.7.4. Each circle represents a substitution model, and the size of each circle represents the degree of support.

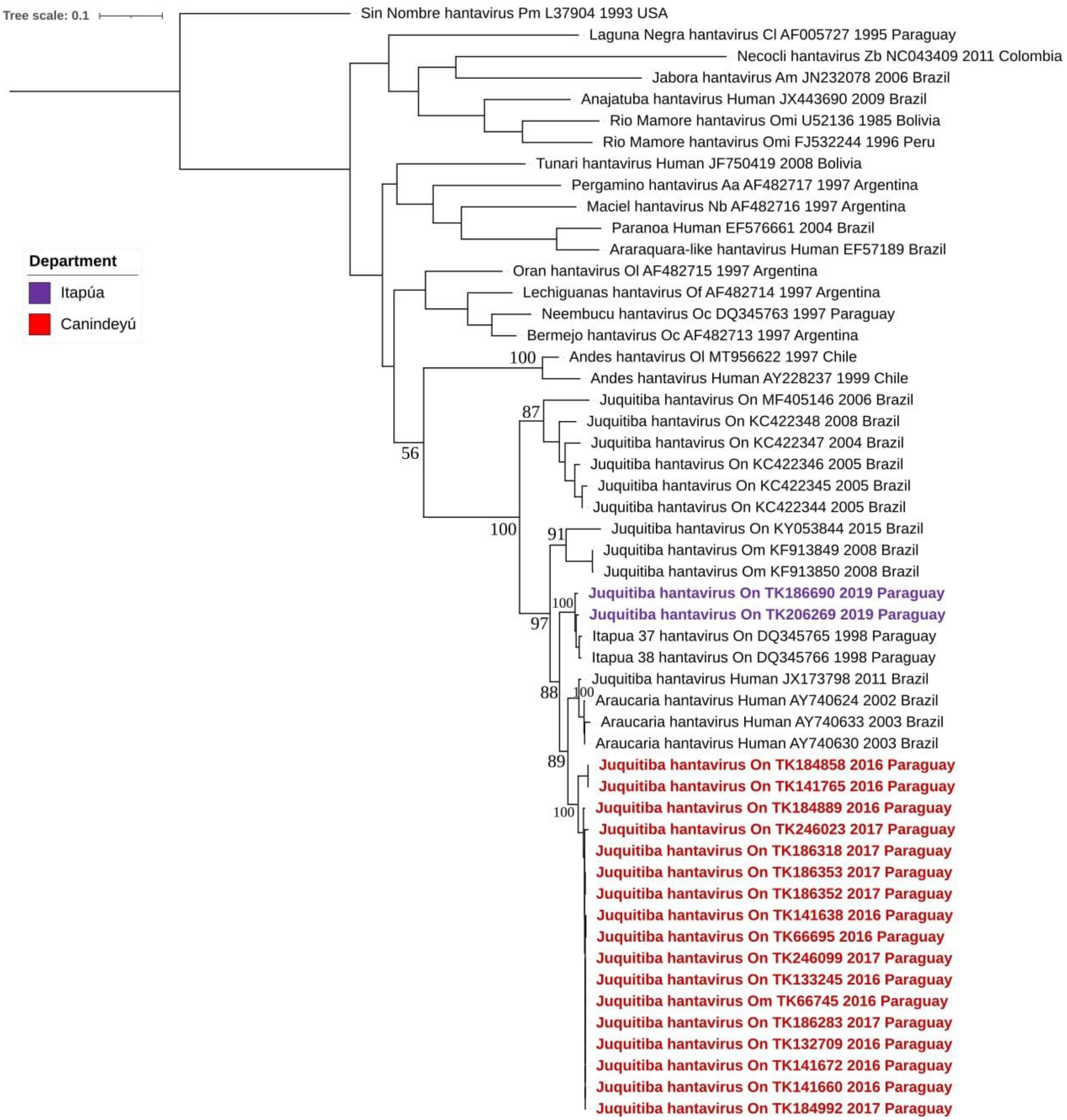

Figure S3. Maximum likelihood phylogenetic analysis of the partial S-segment open reading frame (270-1329 nucleotide regions) of New World hantaviruses. Tree was obtained using the General Time Reversible Model with empirical base frequencies, invariable sites, and gamma distribution with four rate categories (GTR+F++I+G). the evolutionary distance of 0.1 substitutions per position in the sequence. The number of each node indicates the bootstrap values that supported the interior branch. Branch labels include the viral species or strain, the organism that the sequence was obtained from, GenBank accession number or TK number (for samples used in this study), and the year of collection if available. The rodents are indicated by abbreviations: Aa =*Akodon azarae*, Am=*A. montensis*, Cl =*Calomys laucha*, Oc =*O. chacoensis*, Of =*O. flavescens*, Ol =*O. longicaudatus*, Om =*O. mattogrossae*, Omi =O. microtis, On =*O. nigripes*, Nb =*Necromys benefactus*, Pm =*Peromyscus maniculatus*, Zb =*Zygodontomys brevicauda*.

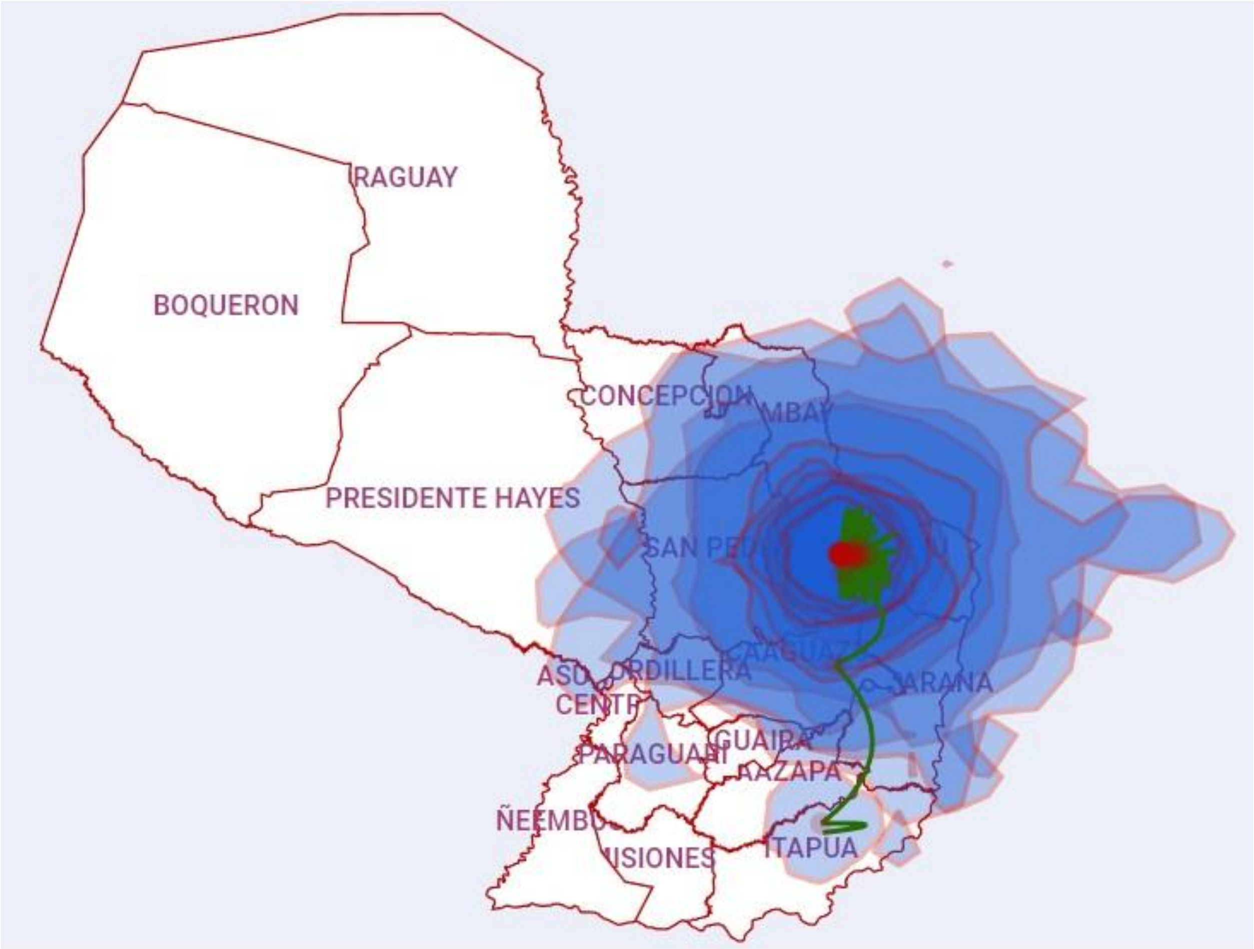

Figure S4. Spatial spread visualization for the S segment. Figure was obtained by uploading the maximum clade credibility tree output from Tree Annotator and the raw posterior tree files. Annotated tree was used to make show spatial divergence, and the raw posterior tree was used to compute the density intervals around the MCC tree. Red dots represent nodes, blue shades represent the height posterior intervals (HPD), and green lines are used to show divergence.

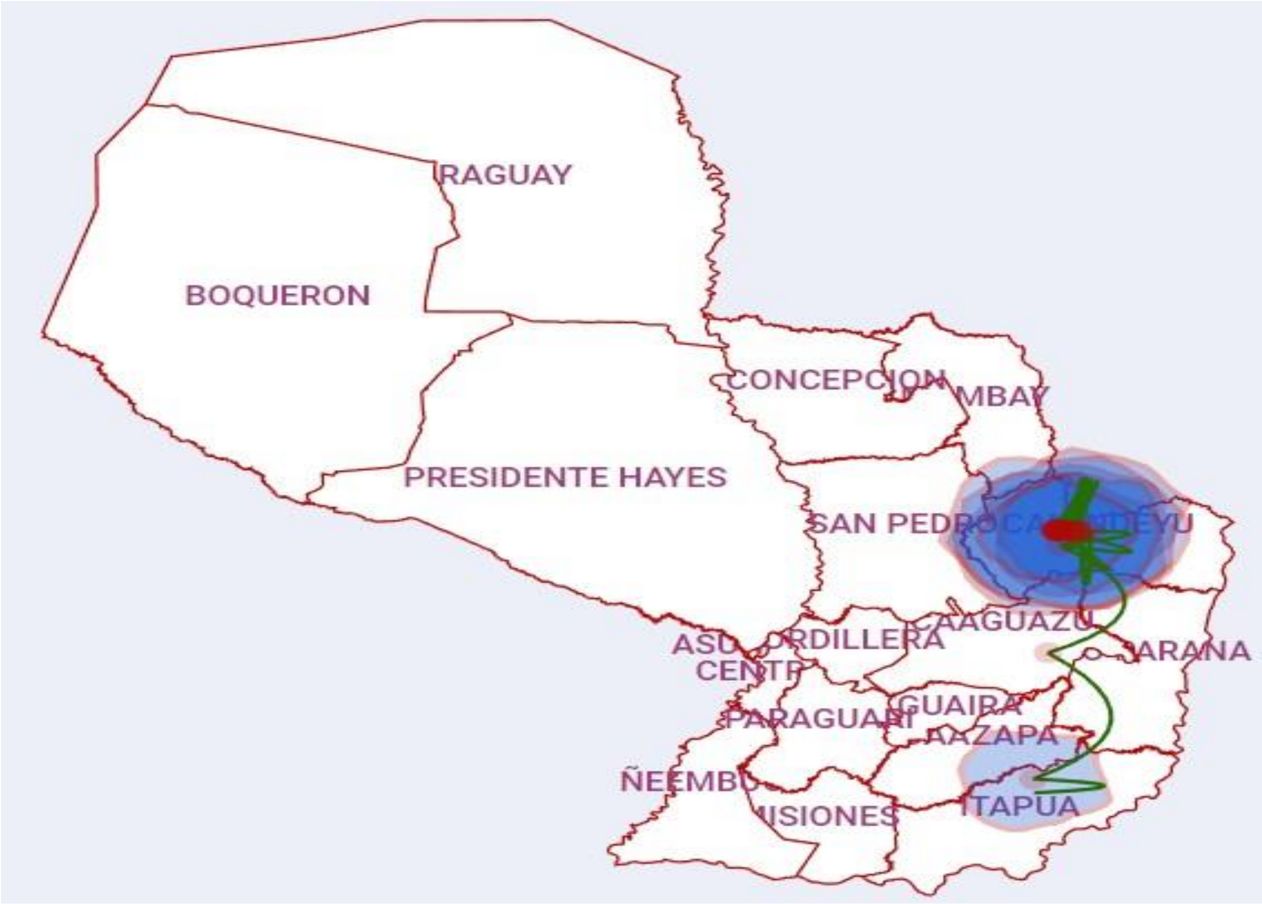

Figure S5. Spatial spread visualization for the M segment. Figure was obtained by uploading the maximum clade credibility tree output from Tree Annotator and the raw posterior tree files. Annotated tree was used to make show spatial divergence, and the raw posterior tree was used to compute the density intervals around the MCC tree. Red dots represent nodes, blue shades represent the height posterior intervals (HPD) for the nodes, and green lines are used to show divergence.

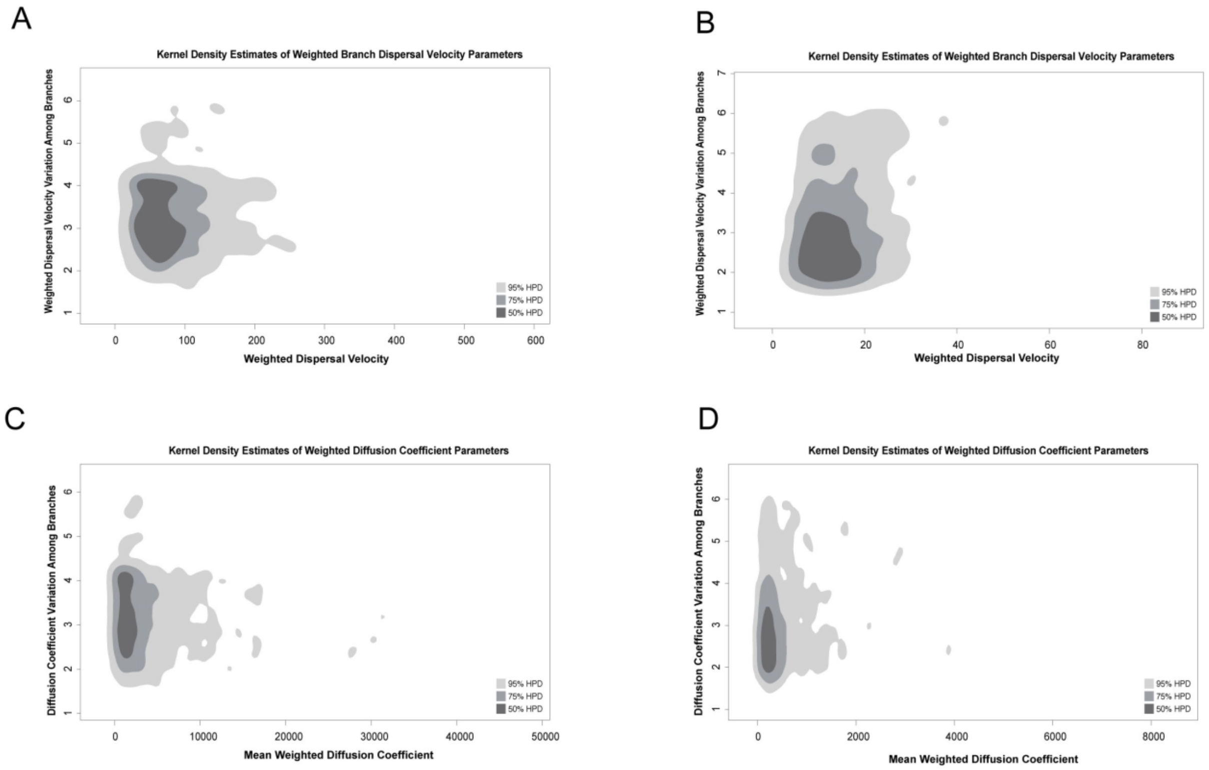

Figure S6. Summary of estimated dispersal statistics. Graphical Illustrations were obtained using the 'spreadStatistics' function of the package 'seraphim' in R (v4.2.3). Weighted branch dispersal velocity for (**A**) S segment, and (**B**) M segment. Weighted diffusion coefficient for (**C**) S segment, and (**D**) M segment. Shades represent the different HPD intervals.

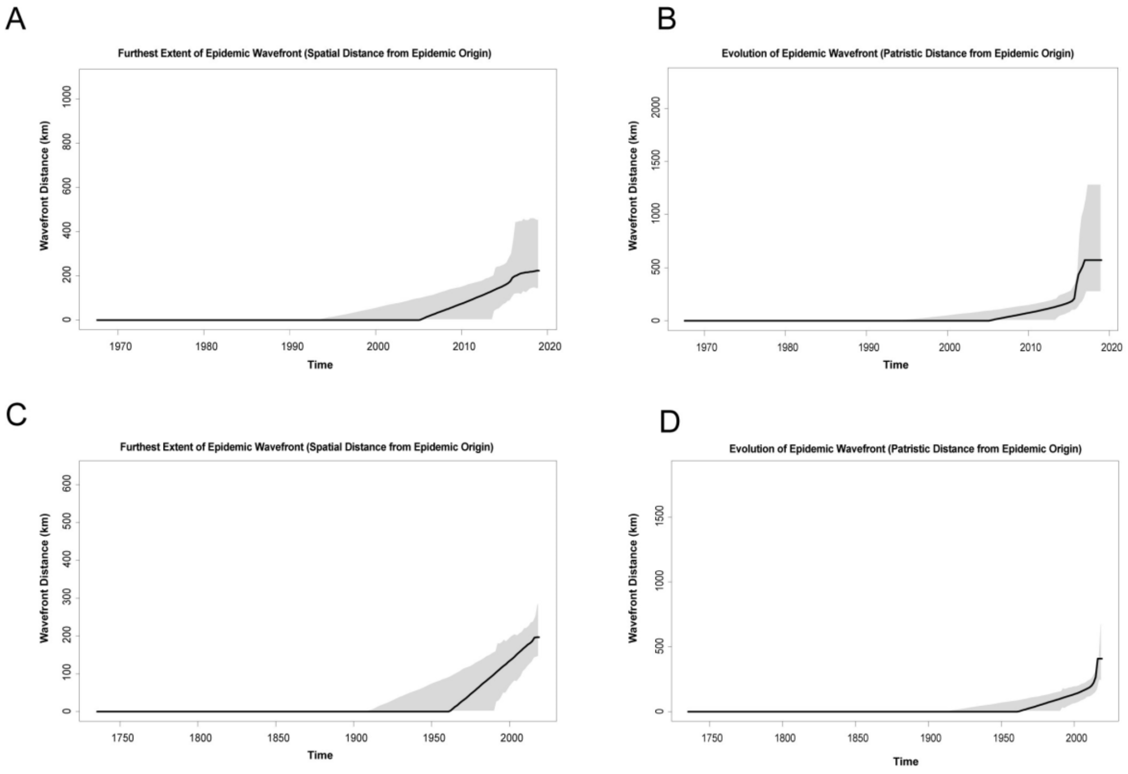

Figure S7. Summary of estimated dispersal statistics (Epidemic wavefronts). Graphical Illustrations were obtained using the 'spreadStatistics' function of the package 'seraphim' in R (v4.2.3). S segment's (**A**) Spatial and (**B**) patristic distances from epidemic origins. M segment's (**C**) Spatial and (**D**) patristic distances from epidemic origins. Gray shades represent 95% credible regions of the estimated wavefront position.
