# Supplementary material for: Deep Sequencing to Reveal Phylo-Geographic Relationships of Juquitiba Virus in Paraguay
Source: Viruses. 2023 Aug 24;15(9):1798. doi: 10.3390/v15091798 (PMC10537311; doi:10.3390/v15091798)
Supplement: Supplementary file 1 [file viruses-15-01798-s001.zip › Supplementary Tables.pdf]

Table S1A: Forward (Pools-F) primer sequences and pool designations for JUQV nucleotide sequences

Pool-F	Name	Primer Sequence	Region	Segment
1F-1	JUQV_S F1	TAG TAG TAG ACT CCT TGA GAA GCT ACT ACT GC	1-32F	S
1F-2	JUQV_S F687	CAT AAG TCC TGT MAT GGG AGT CAT TGG ATT C	687-717F	S
1F-3	JUQV_S F1370	GCT TGA GCC AAT CAG TTA YTC ATC TCA GG	1370-1398F	S
1F-4	JUQV_M F1	TAG TAG TAG ACT CCG CAA GAA GAA GCA AAA AC	1-32F	M
1F-5	JUQV_M F713	CTG ACC ATC TTA AGA TAA TTA GTA CAT TTG AGG G	713-746F	M
1F6	JUQV_M F1324	CTT TAT CTG CCA GAG GGT CGA CCA AGA TG	1324-1352F	M
1F-7	JUQV_M F2054	GGT GAG GTT CCT ATG AAG ACA GAT TTG GAA C	2054-2084F	M
1F-8	JUQV_M F2679	GTG ATA TAA TGT CTA CAA CTG CAG GCA TGA GAT G	2679-2712F	M
2F-1	JUQV_S F262	CTG GCT ACA AAA CCA GTT GAT CCA ACA GG	262-290F	S
2F-2	JUQV_S F910	GAA TCT GCA ACA YTG TTT ACT GAC ATT GCA AC	910-941F	S
2F-3	JUQV_S F1469	GGT TTA TGG GCC ACT ACT GTT CTT ATT TG	1469-1497F	S
2F-4	JUQV_M F271	CTT CAG AAG AGC TAT ACA CAG GTA GAA TGG AG	271-302F	M
2F-5	JUQV_M F868	GTA AGC CGC ATG ATA GTA CAC CCT AGA GG	868-898F	M
2F-6	JUQV_M F1592	GTR TTA ACY TTT TCA TGC TCC CAT TAC ACT AAT GAG TC	1592-1629F	M
2F-7	JUQV_M F2226	GGA TGG ATG CGA CAT TTA ACA TCA AGA CTG	2226-2255F	M
2F-8	JUQV_M F2929	CCG TGA TGT ATC TTT CCA GGA TTT AAG TGA TAA TCC	2929-2964F	M
3F-1	JUQV_S F454	CTG TAC ATG CTT TCA ACC CGA GGG	454-477F	S
3F-2	JUQV_S F1228	CAT TTG GGA GAT GAT ATG GAC CCA GAA CTC AG	1228-1259F	S
3F-3	JUQV_M F412	GAC ACA CTC AAG AGG ATT AAG AAG ACA GTG C	412-442F	M
3F-4	JUQV_M F1153	CCA ATT GCA GGT GAG ATG GAA AAG GTC AC	1153-1181F	M
3F-5	JUQV_M F1770	GTC CCT ATT GTA TGA CCA TGA CTG AAT CTA CAG	1770-1802F	M
3F-6	JUQV_M F2531	CTT GTR ACA CCT TCT GTA AAG GTC TGC A	2531-2557F	M
3F-7	JUQV_M F3190	GTG TTG TCA TGA TAC TGA TTG TTC ATC TG	3190-3218F	M
4F-1	JUQV_S F1	NAGTAGTAGACTCCTTGAGAAGCTACTACTGC	1-32F	S
4F-2	JUQV_M F1	TAG TAG TAG ACT CCG CAA GAA GAA GCA AAA AC	1-32F	M
5F-1	JUQV_S F910	GAA TCT GCA ACA YTG TTT ACT GAC ATT GCA AC	910-941F	S
5F-2	JUQV_M F2679	GTG ATA TAA TGT CTA CAA CTG CAG GCA TGA GAT GC	2679-2713F	M

Table S1B: Reverse (Pools-R) primer sequences and pool designations for JUQV nucleotide sequences

Pool-R	Name	Primer Sequence	Region	Segment
1R-1	JUQV_S R534	GGA TGA GTC ATC TTT AAA CCT GAT CCT AGT CC	503-534R	S
1R-2	JUQV_S R1260	CCT GAG TTC TGG GTC CAT ATC ATC TCC	1235-1260R	S
1R-3	JUQV_S R1869	GCA TTA TTG AGG TAG TAT TGT TGA GGT AGT TAA GG	1835-1869R	S
1R-4	JUQV_M R542	CTA ACC ATA CAG CTT CGT ATT GAC ATA CAT GTG G	509-542R	M
1R-5	JUQV_M R1248	CCA TTC TCA GAA TAT GCT TCA CAA CTT GCT CC	1217-1248R	M
1R-6	JUQV_M R1853	GGA ACC TGC CTG TCA ATT TAC AGA TGG C	1826-1853R	M
1R-7	JUQV_M R2557	GCA GAC CTT TAC AGA AGG TGT YAC AAG RC	2529-2557R	M
1R-8	JUQV_M R3275	CGA TCT GGT TAA AAC CTG TAA CTC GTT CAA GG	3244-3275R	M
2R-1	JUQV_S R829	CCT TAT TTG TTG CCA TGA ATG CCT CAG ATG C	799-829R	S
2R-2	JUQV_S R1371	GAT CCT GAG ATG AAT AAC TGA TTG GCT CAA G	1371-1401R	S
2R-3	JUQV_S R1902	TAG TAG TAT GCT CCT TGA AAA GCA ATC AAG	1873-1902R	S
2R-4	JUQV_M R839	CTA GGT ACA TGC AAT GGT TCT GAA TGA CTT CC	808-839R	M
2R-5	JUQV_M R1472	CCA ATG AAT GGG CAA CAT CAG GCA TC	1447-1472R	M
2R-6	JUQV_M R2142	GGC AGG ATT AGT TAA TTT CCG CCT GTA GC	2118-2146R	M
2R-7	JUQV_M R2813	CCA TCA TTC TCT TAT ACC CTG ACA CAG TAT TGC	2781-2813R	M
2R-8	JUQV_M R3443	GAG GGC ATA GGA GGC TGA ACA AGA TTA TAG	3414-3443R	M
3R-1	JUQV_S R965	GCA AAT ACC CAT ACA GAA KGT GGT GTT G	938-965R	S
3R-2	JUQV_S R1823	GGA TGA GGT AGT ATT GTT GTG ATG TTA GTA CAG C	1790-1823R	S
3R-3	JUQV_M R997	CTT TCA TAG TGT CTG TTG AAC TAG TAC TAG G	966-997R	M
3R-4	JUQV_M R1629	GAC TCA TTA GTG TAA TGG GAG CAT GAA AAR G	1599-1629R	M
3R-5	JUQV_M R2222	GCC CCA ATG GTT GGA TTT CAG CAT GAA TG	2194-2222R	M
3R-6	JUQV_M R3116	GTC GAC CCA TAA CAC ATT GCC AAG TCA C	3082-3116R	M
3R-7	JUQV_M R3674	TAG TAG TAT GCT CGC AGG AAC AAA ATC C	3647-3674R	M
4R-1	JUQV_S R965	GCA AAT ACC CAT ACA GAA KGT GGT GTT G	938-965R	S
4R-2	JUQV_M R1004	GCA GTT CCT TTC ATA GTG TCT GTT GAA CTA G	974-1004R	M
5R-1	JUQV_S R1904	TAG TAG TAT GCT CCT TGA AAA GCA ATC AAG	1875-1904R	S
5R-2	JUQV_M R3674	TAG TAG TAT GCT CGC AGG AAC AAA ATC C	3647-3674R	M

Table S2. S segment hantavirus reference sequences.

Hantavirus	Genbank Accession Codes	Country	Collection Year	Reference
Sin nombre	L37904	USA	1993	PMID: 7494336
Juquitiba	KC422344	Brazil	2005	PMID: 23751399
Juquitiba	KC422345	Brazil	2005	PMID: 23751399
Juquitiba	KC422346	Brazil	2005	PMID: 23751399
Juquitiba	KC422347	Brazil	2004	PMID: 23751399
Juquitiba	KC422348	Brazil	2008	PMID: 23751399
Juquitiba	KY053844	Brazil	2015	Unpublished
Juquitiba	MF405146	Brazil	2006	PMID: 28856421
Juquitiba	KF913850	Brazil	2008	Unpublished
Juquitiba	KF913849	Brazil	2008	Unpublished
Juquitiba	JX173798	Brazil	2011	Unpublished
Araucaria	AY740630	Brazil	2003	PMID: 15977228
Araucaria	AY740624	Brazil	2002	PMID: 15977228
Araucaria	AY740633	Brazil	2003	PMID: 15977228
Itapua 38	DQ345766	Paraguay	1998	PMID: 17172380
Itapua 37	DQ345765	Paraguay	1998	PMID: 17172380
Andes	AY228237	Chile	1999	PMID: 14513715
Andes	MT956622	Chile	1997	Unpublished
Bermejo	AF482713	Argentina	1997	PMID: 11907216
Neembucu	DQ345763	Paraguay	1997	PMID: 17172380
Lechiguanas	AF482714	Argentina	1997	PMID: 11907216
Oran	AF482715	Argentina	1997	PMID: 11907216
Araraquara P5/Cajuru	EF571895	Brazil	Unknown	PMID: 19001829
Paranoa	EF576661	Brazil	2004	PMID: 19103306
Maciel	AF482716	Argentina	1997	PMID: 11907216
Pergamino	AF482717	Argentina	1997	PMID: 11907216
Tunari	JF750419	Bolivia	2008	PMID: 22515983
Rio Mamore	FJ532244	Peru	1996	PMID: 20687859
Rio Mamore	U52136	Bolivia	1985	PMID: 9311652
Anajatuba	JX443690	Brazil	2009	PMID: 23055565
Jabora	JN232078	Brazil	2006	PMID: 22510841
Necocli	NC043409	Colombia	2011	PMID: 26186516
Laguna Negra	AF005727	Paraguay	1995	PMID: 9375015

Table S3. Reciprocal IFA Titers

Species	TK	Collection Site	Collection Date	IFA Titer
Oligoryzomys nigripes	TK186690	Itapúa	02/25/2019	1:128
Oligoryzomys nigripes	TK186704	Itapúa	02/26/2019	1:256
Oligoryzomys nigripes	TK206269	Itapúa	26/26/2019	1:8192

Table S4. Summary of reads, sequencing depths and coverage for the S and M-segments from selected lung samples.

Sample	S-Segment			M-Segment		
	Total Reads	Average Coverage	Coverage (%)	Total Reads	Average Coverage	Coverage (%)
TK186690	107,912	7,680	100	225,868	8,622.91	100
TK206269	75,879	6,668.96	99	212,025	10,665	100
TK206284	1018	57.73	100	1656	42.89	95
TK186704	616	29.31	75	203	4.58	84
TK186673	512	18.74	77	1271	22.87	87

Table S5. Sample numbers and GenBank accession numbers used for phylogenetic and phylogeographic analyses of S and M segments.

Department	Sample	GenBank Accession Numbers	
		S Segment	M Segment
Itapúa	TK186690	OR184117	OR184122
	TK206269	OR184118	OR184123
	TK184992	OR184959	OR184986
	TK66695	OR184961	OR184976
	TK132709	OR184962	OR184978
	TK133245	OR184963	OR184979
	TK141638	OR184964	OR184980
	TK141660	OR184965	OR184981
	TK141672	OR184966	OR184982
	TK141765	OR184967	OR184983
Canindeyú	TK184858	OR184968	OR184984
	TK186283	OR184969	OR184987
	TK186318	OR184970	OR184988
	TK186352	OR184971	OR184989
	TK186353	OR184972	OR184990
	TK246023	OR184973	OR184991
	TK246099	OR184974	OR184992
	TK184889	OR184975	OR184985
	TK66745	OR184691	OR184977
